# Region Based CNN for Foreign Object Debris Detection on Airfield Pavement

**DOI:** 10.3390/s18030737

**Published:** 2018-03-01

**Authors:** Xiaoguang Cao, Peng Wang, Cai Meng, Xiangzhi Bai, Guoping Gong, Miaoming Liu, Jun Qi

**Affiliations:** 1 Image Processing Center, Beijing University of Aeronautics and Astronautics, Beijing 100191, China; xgcao@buaa.edu.cn (X.C.); paulwp@buaa.edu.cn (P.W.); gonggp@buaa.edu.cn (G.G.); liumiaoming@ime.ac.cn (M.L.); qijun1994@126.com (J.Q.); 2 State Key Laboratory of Virtual Reality Technology and Systems, Beijing University of Aeronautics and Astronautics, Beijing 100191, China

**Keywords:** foreign object debris, object detection, convolutional neural network, vehicular imaging sensors

## Abstract

In this paper, a novel algorithm based on convolutional neural network (CNN) is proposed to detect foreign object debris (FOD) based on optical imaging sensors. It contains two modules, the improved region proposal network (RPN) and spatial transformer network (STN) based CNN classifier. In the improved RPN, some extra select rules are designed and deployed to generate high quality candidates with fewer numbers. Moreover, the efficiency of CNN detector is significantly improved by introducing STN layer. Compared to faster R-CNN and single shot multiBox detector (SSD), the proposed algorithm achieves better result for FOD detection on airfield pavement in the experiment.

## 1. Introduction

After a plane crashed in Paris de Gaulle, the detection of foreign object debris (FOD) on airfield pavement has become more and more important. Actually, FOD detecting is one of the crucial technologies for intelligent vehicular systems. The safety and convenience of modern transportation could be significantly improved by adopting an efficient FOD detecting system. However, complex airfield environment makes the detection of FOD a challenging task. Due to the variation of background and influence of automotive imaging system, FODs could be hardly detected and recognized on airfield pavement by traditional features, such as scale invariant feature transform (SIFT) [1], histograms of oriented gradients (HOG) [2] and local binary patterns (LBP) [3].

Deep learning is one of the rapidly developing technologies in the area of big data. Since [4] significantly improved the effect of image classification, convolutional neural network (CNN) has been widely introduced into computer vision applications, such as image classification [5,6,7,8], face verification [9,10,11], semantic segmentation [12,13,14,15], object detection [16,17,18] and image annotation [19,20,21]. Moreover, some CNN based algorithms are proposed to solve transportation problems [22,23,24]. Based on various public available datasets such as ImageNet [25], Pascal VOC [26] and COCO [27], CNN algorithms have been proved to perform better in detection and recognition than traditional feature methods. Compared with these manually designed features, CNN based features have better resolution and robustness for FOD detection. Actually, the FOD problem consists of two tasks: target location and object classification on pavement. Aimed at these two tasks, a novel two-stage framework is designed and introduced in this paper. In the first stage, region proposal network (RPN) [28], as a kind of fully convolutional network (FCN) [12], is trained end-to-end to generate FOD location proposals. In the second stage, CNN classifier based on spatial transformer network (STN) [29] is applied to obtain the parameters of scale, rotation and warping. Due to the performance of STN, FODs could be correctly identified by generated features, regardless of image distortion.

A preliminary version of this paper was presented in [30]. The highlights and extensions in this paper could be summarized as follows.

1. A new FOD detection framework based on CNN models for FOD detection is proposed with improved region proposal network and spatial transformer network.
2. RPN is firstly introduced and improved to generate high quality region proposals for FOD detection on airfield pavement. In addition, some candidate select rules are designed to reduce quantity and improve quality of region proposals.
3. The STN based CNN classifier is proved to be effective for FOD classification. Moreover, the proposed framework achieves better result than other detection algorithms, such as faster-RCNN [28] and SSD [17], for FOD detection on airfield pavement.
4. The vehicular imaging system, including DGPS, cameras, alarm, FOD management system and remote query system, is presented and discussed in detail.

The rest of this paper contains four main sections. In Section 2, some recent works are analyzed, including traditional feature based algorithms and some CNN algorithms. In Section 3, the overall detection framework is given in details, including location based on RPN and classification based on STN. In Section 4, comparison with other algorithms is conducted and discussed. Conclusions are given and discussed in Section 5.

## 2. Related Work

To solve FOD detection problem, some effective algorithms are proposed recently [31,32,33,34,35,36]. The algorithms based on different sensors, such as actively scanning LiDAR system [31], mm-wave FMCW radar [34] and wideband 96 GHz Millimeter-Wave Radar [35], could achieve good results in different environments. A cosecan squared beam pattern in elevation and a pencil-beam pattern in azimuth, generated through folded reflectarray antenna (FRA) by phase only control, is analyzed to detect objects on ground [36]. A multi-sensor system, based on inherent feature of FOD, is introduced to detect and recognize FOD [32]. Based on a large amount of prior knowledge and manually designed feature extractor, these methods could transform pixel values of an image into a suitable internal representation. Thus, these methods are effective for detection of FODs with less noise, but not working for FODs with complex background and noise.

Actually, CNN based detection has becoming more and more popular. There are two basic research methods: region proposal based and non-region proposal based algorithms. Region proposal based algorithm, such as faster R-CNN [28], R-FCN [37] and Mask R-CNN [38], consists of a region of interest (ROI) generator and an object classifier. Region proposal network (RPN) is designed in faster R-CNN [28], by introducing the classification features into ROI extraction module. In this way, better region proposals could be generated in less time. Moreover, this leads to high recognition accuracy and good real-time performance. Semantic segmentation method is introduced in R-FCN [37], which could calculate classification possibility of all region proposals in one image at the same time. This enlightening strategy greatly reduces the running time of ROI classification. Finally, object detection, semantic segmentation and key point detection are solved by one CNN algorithm [38]. Non-region proposal based algorithm, such as YOLO [16] and SSD [17], usually has better real-time performance with lower detection accuracy. Object detection is described as a regression problem in YOLO [16], with an end-to-end network. YOLOv2 and YOLO9000 [39] are two improved versions with faster speed and higher accuracy. However, YOLO could hardly detect small objects such as FODs on pavement. Similar to YOLO, SSD [17] is also based on regression of target bounding box. To achieve higher accuracy, feature maps from multi-layers are introduced to the locating and scoring module. This strategy has been proved to achieve better performance.

To detect FODs on airport pavement, improved RPN and STN based CNN classifier are designed as given in Section 3.

## 3. Algorithm

**Figure 1:**
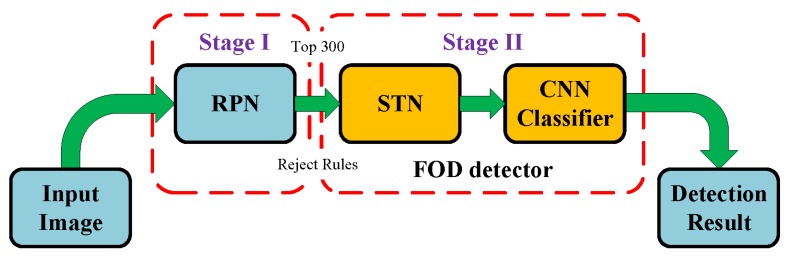
The framework of the FOD detection in this paper.

The FOD detection framework is shown in Figure 1, which contains two stages. Firstly, region proposal network (RPN) [28] is adopted to generate a set of original object proposals, which are taken as FOD candidates. Moreover, some select rules are designed to reduce computational cost and fix image size. Secondly, spatial transformer network (STN) [29] is introduced to adjust the targets in region proposals from the influence of scale, rotation and warping. Then, these adjusted proposal images are fed to CNN classifier to extract features and identify FODs.

### 3.1. Locate FOD Candidates with Improved RPN

Generally, sliding window on images is a widely used strategy for target detection. With well-designed features, such as SIFT [1] and HoG [2], the window with target inside could be identified from background. In this way, the target detection problem could be described as a classification problem in sliding windows. However, sliding window is a low efficiency method, which costs much computing time and memory. Recently, a series of region-based convolutional neural networks (R-CNN) [28,37,38,40,41] is proposed to detect targets using deep learning method. One of the most meaningful ideas from R-CNN algorithms is the region proposal strategy to locate target candidates from background. On the one hand, region proposal algorithm is much faster than sliding window on whole image because region proposal algorithm reduces the number of candidates from millions to hundreds or thousands. On the other hand, region proposal algorithm has higher recall rate for finding all objects in an image, which could improve object location efficiency significantly. In this paper, some prior knowledge is introduced in region proposals to reduce the number of FOD candidates for better location.

**Figure 2:**
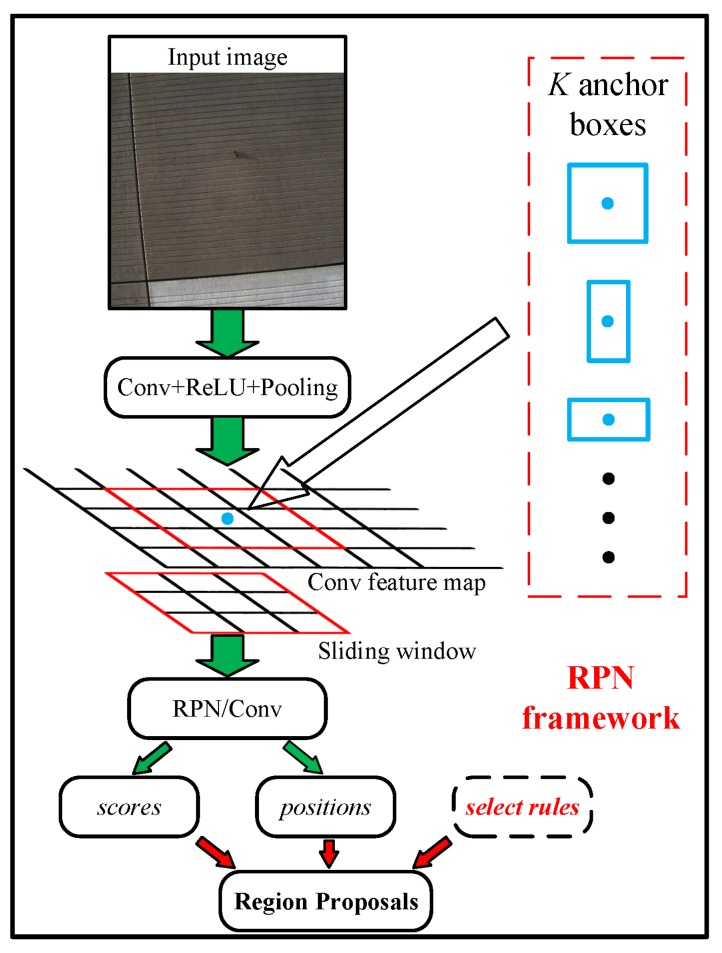
The RPN framework for FOD location.

The algorithm to generate region proposals is based on region proposal network (RPN) [28]. Actually, RPN takes an image as input and generates a series of rectangular object candidates with corresponding objectness scores. To generate region proposals as shown in Figure 2, a small network is slid over the convolution feature map. In each sliding window, *k* reference boxes (anchors) [28] is defined in different scales and aspect ratios to predict various region proposals. In other words, RPN is designed with the help of convolution features from detection module. The intersection over union (IoU) between region proposals and ground-truth bounding box, is chosen as evaluate index of the proposed FOD detection framework. For FOD detection in our dataset, targets in average shape 80×80 locate in 2048×2048 input image. To match the scale of FODs, the anchor boxes are set with area of $100^{2}$ and aspect ratios of 1:1, 1:2 and 2:1.

Although there are much fewer FOD candidates generated by RPN than by common sliding window paradigm, the candidate number is still very large for classification. To further reduce FOD candidates, a novel series of select rules is designed and introduced in RPN framework. Actually, candidate FOD bounding boxes by original RPN vary in different sizes. For all region proposals, FODs such as screws and stones have different shape scales from false alarms. Therefore, three select rules based on prior knowledge are proposed and listed as below.

**Rule 1:** Region proposals by RPN are filtered by high aspect ratio Ratio, which is expressed as

(1) $$\begin{array}{c} Ratio=\frac{\max(w,h)}{\min(w,h)},\\ Ratio<T_{ratio}. \end{array}$$

*w* and *h* are the width and height of the image, respectively, and $T_{ratio}$ is a threshold with a constant value.

**Rule 2:** The areas of proposals containing FODs should be in range [$T_{min},T_{max}$], which is expressed as

(2) $$\begin{array}{c} Area=w\times h,\\ T_{min}\leq Area\leq T_{max}. \end{array}$$

Area is the area of region proposal.

**Rule 3:** The proposals with higher objectness scores $O_{l}$ generated by RPN are picked out, which is expressed as

(3) $$O_{l}>T_{objectness}.$$

$T_{objectness}$ is a constant threshold for judging objectness.

The thresholds for these three rules are set as $T_{ratio}=1.5$, $T_{min}=60^{2}$, $T_{max}=100^{2}$ and $T_{objectness}=0.8$. By deploying these rules, the number of region proposals by improved RPN could be greatly reduced by about 60%. In this way, more accurate candidate proposals with fewer number could be generated. Moreover, the efficiency of FOD classification could be greatly improved.

### 3.2. Spatial Transformer Network

**Figure 3:**
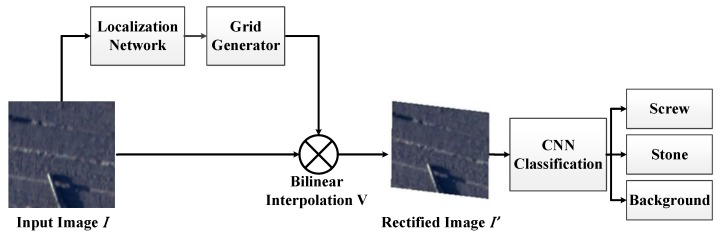
The FOD classification framework.

**Figure 4:**
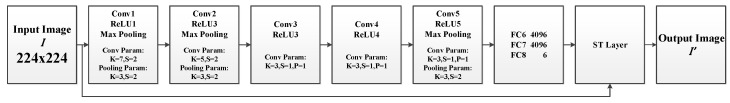
The architecture of spatial transformer network.

STN [29] is an effective CNN framework to learn scale, rotation and warping of images. With a predicted affine transformation by STN, an input image I(x,y) could be adjusted to a rectified image $I^{\prime }\left(x,y\right)$. As shown in Figure 3, affine matrix is firstly regressed via a localization network. Then, predicted transformation parameters are used to generate a sampling grid. The sampling grid is a set of points from input map for affine transformation. Finally, feature map and the sampling grid are utilized as inputs to be sampled. The architecture of STN is shown in Figure 4. Details of STN algorithms are given below.

#### 3.2.1. Localization Network

The localization network takes feature map with width *w*, height *h* and channel *c* as input *U*. Then, the transformation parameters could be generated by $O=f_{loc}\left(U\right)$. In this paper, the effect of vehicular imaging system is assumed as affine transformation. Therefore, output of localization network *O* is a six-dimensional vector from fully-connect layer. Actually, there are five convolution layers and three fully-connect layers in the localization network.

#### 3.2.2. Grid Generator

To perform a warping of input feature map, each output pixel is computed by locating a sampling kernel in a particular area of the input feature map. In general, the output pixels are defined on a regular grid $G_{i}=\left(x_{i}^{t},y_{i}^{t}\right)$, which form an output feature map *P*. The relationship between input and output feature maps is expressed as below.

(4) $$\left[\begin{array}{c} x_{i}^{s}\\ y_{i}^{s} \end{array}\right]=\Gamma_{\theta }\left[\begin{array}{c} x_{i}^{t}\\ y_{i}^{t}\\ 1 \end{array}\right]=\left[\begin{array}{ccc} \theta_{11} & \theta_{12} & \theta_{13}\\ \theta_{21} & \theta_{22} & \theta_{23} \end{array}\right]\left[\begin{array}{c} x_{i}^{t}\\ y_{i}^{t}\\ 1 \end{array}\right]$$

In Equation (4), $\Gamma_{\theta }$ signifies affine transformation matrix for image distortion.

#### 3.2.3. Sampler

In the sampler module, pixels in $P^{\prime }$ are calculated through regional bilinear interpolation on the input image *P*. With all pixel values obtained, the rectified image *I* could be generated as:

(5) $$I^{\prime }=V\left(P,I\right)$$

*V* represents the bilinear interpolation. Specifically, *V* is also a differentiable module for back propagation.

### 3.3. FOD Classification with Convolutional Neural Network

**Figure 5:**
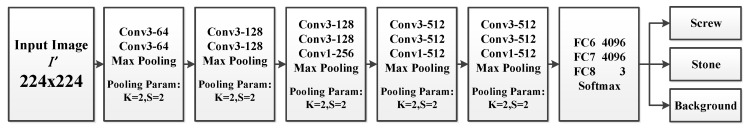
The FOD classification architecture.

In FOD classification framework, each extracted region proposal is identified as background, stone or screw. A convolutional neural network (CNN) classifier is adopted for this application, as presented in Figure 5. The network is based on the type C of visual geometry group (VGG) model [5], which is one of the most famous CNN algorithm. There are thirteen convolution layers followed by three fully-connect layers in VGG model. The FOD classification network is fine-tuned from pre-trained models of VGG on ILSVRC2012 dataset [25], with the Caffe toolbox [42] in our experiments.

Actually, the last layer of VGG model is a fully-connect layer with 1000 output neurons. To match our application with the pre-trained VGG model, the first fifteen layers of VGG model are taken as feature extractor. The last layer of VGG model is replaced by a classifier to predict the candidate rectangular regions as background, screw or stone. Therefore, only three output neurons are necessary in the last layer of classification network.

## 4. Experiments

**Figure 6:**
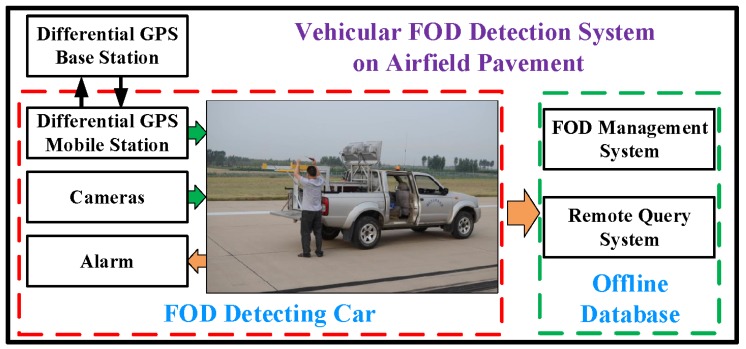
The framework of vehicular FOD detection system on airfield pavement.

The airfield pavement images for experiment are sampled by a vehicular imaging system in Tianjin Binhai International Airport (ZBTJ). Actually, there are already some automatic vehicular imaging and processing systems, such as PCES, PAVUE and ARAN. The framework of vehicular FOD detection system on airfield pavement in this paper is shown in Figure 6. There are mainly two parts: FOD detecting car and offline database in this framework. In detail, some important modules are listed as below.

**DGPS:** The differential GPS (DGPS) base station and mobile station could provide the real-time position of the FOD detecting car and the detected FODs.

**Cameras:** There are four GT2050C cameras with 2048×2048 resolution scanning for 5m in width at the same time.

**Alarm:** The information of predicted FODs, including FOD classes and accurate locations from the image and DGPS, is sent as alarm and saved in offline database.

**FOD Management System:** The detection and cleaning of FODs are managed and upgraded by the management system in offline database.

**Remote Query System:** The real-time status of FODs in database could be remotely inquired by others, such as airfield pavement cleaning system and plane operating system.

To meet the 25 fps real-time sampling frequency, the speed of FOD detection car should be less than 31.25 m/s and the processing time for each frame should be less than 40 ms. With GTX 1080ti gpu, the proposed detection algorithm could achieve 26 fps with high accuracy, which is faster than the original faster R-CNN [28] with 14 fps and slower than SSD [17] with 31 fps.

For FODs on pavement, lamp covers, marker lines, dilapidations and tire marks are the main noise and false alarms. This makes FOD detection a complex and challenging task. Actually, screws and stones are major targets to be recognized for airfield pavement FOD detection. These two kinds of FODs and background make up the final three predict classes.

### 4.1. The Dataset and Training

**Figure 7:**
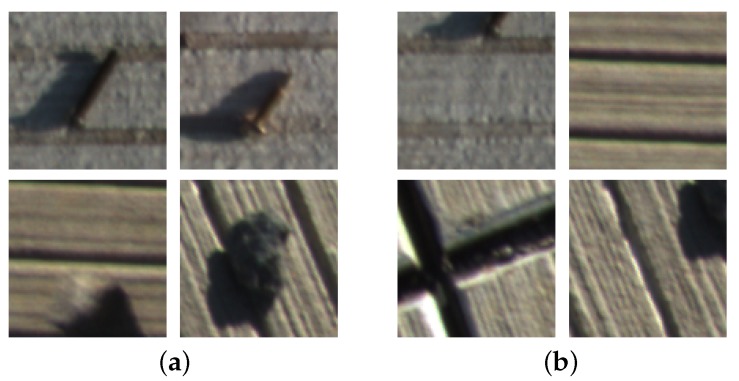
(**a**) Positive FOD samples with IoU ≥ 0.7; and (**b**) negative FOD samples with IoU ≤ 0.3.

The airfield pavement image dataset contains 12,231 images with shape 2048×2048 sampled by the vehicular imaging system in our dataset. In these images, there are 3562 screws and 4202 stones. The number of foreign object debris in one image varies from zero to four. The shape of boundary box for screws and stones is 80×80 on average, in the dataset ground truth. Region proposals by RPN are employed to fine-tune the FOD detector with STN. The region proposals with IoU ≥ 0.7 over ground-truth boxes are treated as positive, while the region proposals with IoU ≤ 0.3 are labeled as negative, as shown in Figure 7. The top 100 score region proposals are chosen as training samples. This makes the number of negative samples much larger than that of positive samples in our application. To obtain a reasonable number of candidates, 16 stone windows, 16 screw windows and 32 background windows are used in each training batch.

To train the FOD Detector, 60% of images are taken as training dataset, another 20% are taken as validate dataset and the rest for test. The Caffe Toolbox [42] are chosen to train our networks, with Stochastic Gradient Descent (SGD) [43] for optimization. As one of the most widely used CNN models, VGG model for fine-tuning is trained on 1.2 million images from ILSVRC2012 dataset with 1000 classes. Before fine-tuning, parameters of all layers except FC8 layer are initialized with the pre-trained type C VGG model. In this way, the training time could be apparently reduced and a better fine-tuned model could be generated. The number of training iterations is set as 50,000. The learning rate is initially set as 0.001 and decaying by 0.1 after each 10,000 iterations.

### 4.2. The Experiments of Location

**Figure 8:**
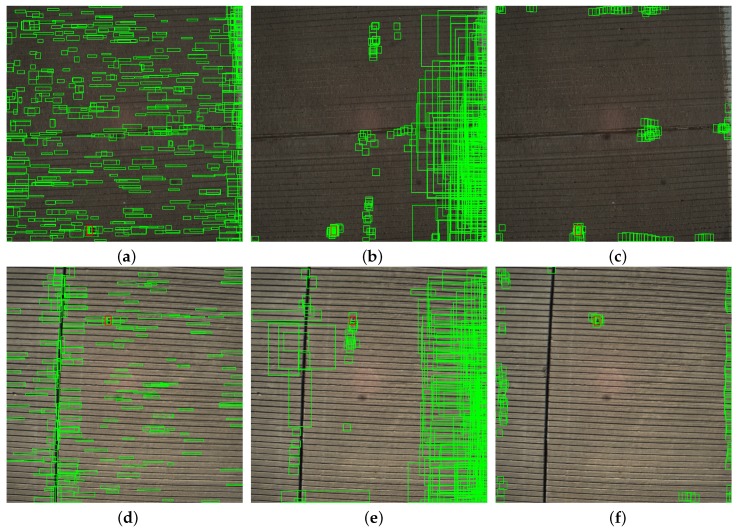
(**a**,**d**) FOD location results by Selective Search; (**b**,**e**) results of original RPN; and (**c**,**f**) results of improved RPN. The green boxes are generated FOD candidates by these two methods respectively, while red boxes are ground truths.

Due to the sequential structure of the most target detection algorithms, location efficiency significantly affects classification results. Candidates with more accurate locations could be more easily classified as targets or background, especially for CNN-based classifier. Actually, RPN is improved by adding prior knowledge based select rules, to obtain FOD candidates with high quality. To evaluate generated proposals, Figure 8 shows the candidate rectangles extracted by Selective Search [40,41,44], original RPN in faster R-CNN [28] and improved RPN for images in our dataset.

The patches generated by Selective Search and original RPN vary in different sizes and may contain multiple objects. This uncertainty affects the accuracy and robustness of FOD detector and produces more false positive results. The improved RPN generates much less region proposals with more accurate locations. To compare the quality and quantity of generated proposals, proposal numbers and recall rates in different IoU levels are shown in Table 1.

In general, the number of candidate boxes generated by RPN is less than that generated by Selective Search, but the recall rate is about 10% higher. It indicates that region proposals by RPN are with high quality and easy to be identified by the followed FOD classifier.

### 4.3. The Experiments of Classification

The STN is proposed to learn and rectify image distortion, such as scale, rotation and warping. Actually, CNN classifier with STN could improve classification accuracy, especially on small dataset. To measure the effect of STN, four comparative experiments are conducted. These four experiments are FOD detector without fine-tune, STN based FOD detector without fine-tune, FOD detector with fine-tune and STN based FOD detector with fine-tune. These results are presented in Table 2.

As presented in Table 2, the recall rates of FOD classification are significantly increased by introducing STN. This is because STN decreases the effect of image distortion while sampling. Compared to ImageNet or Pascal VOC, the sample number is much smaller in our dataset, which the various image distortions may not be fully represented by samples. With fine-tuning from VGG model, STN based FOD classifier could achieve high accuracy for classification.

### 4.4. Comparison with Other Algorithms

To test the performance of proposed method, three other object detection algorithms are introduced for comparison. These algorithms are faster R-CNN [28], Single Shot MultiBox Detector (SSD) [17] and Selective Search [44] with FOD detector. Faster R-CNN is the third generation of the region proposal based CNN algorithms, in which RPN is proposed to enhance the real-time detection performance. RPN is designed to generate region proposals through convolution feature maps instead of original image. With the help of shared features from classification network, RPN could achieve high recall rate with less running time. As region proposals by RPN have good robustness for various target scales, faster R-CNN are one of the most popular detection CNN algorithms. In our experiment, the Zeiler and Fergus model [45] based faster R-CNN is deployed for comparison. SSD, as an end-to-end fully convolutional network, is based on regression of target bounding box. Usually, regression based detection algorithm, such as [16,17], has good real-time performance with lower accuracy, comparing to region based CNNs. To increase the accuracy of location and classification, features from multi-layers are introduced to the locating and scoring module in SSD framework. As one of the most famous regression based detection CNNs, SSD512 model is deployed for comparison with the proposed algorithm. For compared faster R-CNN and SSD, parameters are set as the same to the original algorithms [17,28], except for object class numbers. the Moreover, Selective Search method with the proposed STN based FOD detector is also conducted to evaluate detection efficiency.

For all experiments, if the correctly identified candidate has a greater value than 0.5 intersection over union (IoU) overlap with a ground-truth box, this will be recorded as a successful recall. All wrongly detected candidates, including wrongly sorted targets, are counted as false alarms. In detail, false alarm rate (FAR) and recall rate (RR) are, respectively, defined as:

(6) $$\begin{array}{c} FAR=\frac{TN}{TP+TN},\\ RR=\frac{TP}{TP+FP}. \end{array}$$

In Equation (6), TP is the number of true positive samples, TN is the number of true negative samples, FP represents the number of false positive samples, and FN signifies the number of false negative samples.

FARs of four algorithms are shown in Table 3, while RRs for screws and stones are shown in Table 4. It indicates that the proposed algorithm could predict FODs with the least false alarms and the highest recall rates. Faster R-CNN uses RPN as candidate detection network, which could achieve high recall rates for all classes regardless of FOD scales. However, more backgrounds are also introduced as region proposals. This is because the sample number of dataset is not large enough to train the model with good candidate selecting capability. Although SSD greatly enhances the real-time performance, detection for small objects is not effective. Actually, the average length ratio between FODs and whole images is about 0.04, which is usually considered as small target detection problem [46,47,48,49]. It means that convolution features could possibly be lost through pooling operation with no fully-connect layers. This leads to high FAR and low PR of SSD algorithm for small objects, such as FODs. Selective Search algorithm generates more FOD candidates as shown in Figure 8, which lead to high FAR usually. However, Selective Search with FOD detector achieves low FAR due to the proposed high efficiency classifier. In other words, the proposed STN based classifier correctly identified most of the candidates by Selective Search. Moreover, RRs for both classes are limited by the low recall rate of Selective Search, as shown in Table 1 in Section 4.2.

The proposed algorithm achieves low FAR and high RR due to improved RPN and STN based classifier with high accuracy. Actually, the improved RPN has some extra select rules which could remove more false alarms for FOD detection. The STN based classifier could correctly identify most of the rest region proposals as one of FOD classes or background.

To compare the overall performance of four algorithms, mean average precision (mAP) is calculated according to different precision-recall pairs. The mAPs of different algorithms are listed in Table 5. The proposed algorithm achieves the highest mAP, which means that the proposed algorithm is effective and robust for FOD detection.

These experiments generally indicate that FOD detector with RPN outperforms faster R-CNN, SSD and Selective Search with FOD detector in our dataset. This is because the candidate boxes produced by improved RPN are with better quality and quantity than other detection algorithms. STN based classifier could correctly identify most of the generated region proposals as one of FOD classes or background, regardless of image distortion.

## 5. Conclusions

This paper presents a method based on region proposal network (RPN) and convolutional neural network (CNN) with spatial transformer network (STN) to detect foreign object debris (FOD). Some extra region proposal select rules are introduced to improve the quality and quantity of FOD candidates generated by RPN. Moreover, CNN classifier with STN could learn rotation invariant features from image, which usually are not fully represented by a small number of samples. The experiments indicate that the proposed algorithm is more effective and robust than some popular detection algorithms, such as faster R-CNN and Single Shot MultiBox Detector for FOD detection.

## Figures and Tables

**Table 1:** The recall of Selective Search and RPN.

Methods	IoU	Recall Num	Total Num	Recall Rate	Average Num
Selective Search	IoU > 0.5	2108	2469	85.37%	800
	IoU > 0.6	1875	2469	75.94%	800
Region Proposal Network (RPN)	IoU > 0.5	2263	2469	91.65%	Top5
	IoU > 0.6	2253	2469	91.25%	Top5
	IoU > 0.5	2399	2469	97.16%	Top10
	IoU > 0.6	2394	2469	96.96%	Top10
	IoU > 0.5	2462	2469	99.72%	Top20
	IoU > 0.6	2461	2469	99.60%	Top20

**Table 2:** The results of classification.

FOD Detector	Recall Rate
FOD classification (no fine-tune)	94.52%
**STN + FOD classification (no fine-tune)**	**96.31%**
FOD classification + fine-tune	96.45%
**STN + FOD classification + fine-tune**	**97.67%**

**Table 3:** The detection evaluations by FAR.

Methods	FAR
faster R-CNN	11.02%
SSD	8.19%
Selective Search + FOD Detector	1.21%
**RPN + FOD Detector**	**0.66%**

**Table 4:** The recall rates of screw and stone.

Methods	Screw RR	Stone RR
faster R-CNN	83.51%	93.84%
SSD	87.72%	88.63%
Selective Search + FOD Detector	80.63%	81.46%
**RPN + FOD Detector**	**96.90%**	**96.40%**

**Table 5:** The mean average precisions.

Methods	mAP
faster R-CNN	89.43%
SSD	89.92%
Selective Search + FOD Detector	96.65%
**RPN + FOD Detector**	**98.41%**
